# Small scale homelike special care units and traditional special care units: effects on cognition in dementia; a longitudinal controlled intervention study

**DOI:** 10.1186/s12877-016-0222-5

**Published:** 2016-02-16

**Authors:** Jeroen S. Kok, Marieke J. G. van Heuvelen, Ina J. Berg, Erik J. A. Scherder

**Affiliations:** Lentis, Mental Health Care Institute, PO Box 128, 9470 AC Zuidlaren, The Netherlands; Center for Human Movement Sciences, University Medical Center/ University of Groningen, Groningen, The Netherlands; Department of Clinical Neuropsychology, VU University Amsterdam, van der Boechorstraat 1, 1081 BT Amsterdam, The Netherlands

**Keywords:** Dementia, Cognitive disorders, Neuropsychology, Long term care, Nursing home

## Abstract

**Background:**

Evidence shows that living in small scale homelike Special Care Units (SCU) has positive effects on behavioural and psychological symptoms of patients with dementia. Effects on cognitive functioning in relation to care facilities, however, are scarcely investigated. The purpose of this study is to gain more insight into the effects of living in small scale homelike Special Care Units, compared to regular SCU’s, on the course of cognitive functioning in dementia.

**Methods:**

A group of 67 patients with dementia who moved from a regular SCU to a small scale homelike SCU and a group of 48 patients with dementia who stayed in a regular SCU participated in the study. Cognitive and behavioural functioning was assessed by means of a neuropsychological test battery and observation scales one month before (baseline), as well as 3 (post) and 6 months (follow-up) after relocation.

**Results:**

Comparing the post and follow-up measurement with the baseline measurement, no significant differences on separate measures of cognitive functioning between both groups were found. Additional analyses, however, on ‘domain clusters’ revealed that global cognitive functioning of the small scale homelike SCU group showed significantly less cognitive decline three months after the transfer (*p* < 0.05).

Effect sizes (95 % CI) show a tendency for better aspects of cognition in favour of the homelike small scaled SCU group, i.e., visual memory, picture recognition, cognitive decline as observed by representatives and the clustered domains episodic memory and global cognitive functioning.

**Conclusions:**

While there is no significant longitudinal effect on the progression of cognitive decline comparing small scaled homelike SCU’s with regular SCU’s for patients with dementia, analyses on the domain clusters and effect sizes cautiously suggest differences in favour of the small scaled homelike SCU for different aspects of cognition.

## Background

Dementia is a syndrome that, depending of the type of dementia, can be characterized by a deterioration of several cognitive functions as memory, language, executive functioning, attention, and visuospatial abilities [1–4]. As the dementia progresses patients also show behavioural and neuropsychiatric symptoms as a result of a disturbed understanding of the environment, known as Behavioural and Psychological Symptoms of Dementia (BPSD), as sleep changes, irritability and agitation [5]. BPSD symptoms as apathy, hallucinations, night time agitation and anxiety seem to a considerable extent related to long-term institutionalization [6].

Patients with dementia and extensive neuropsychiatric symptoms are likely to be cared for in Specialized Care Units (SCU’s) [7, 8], developed to meet their specific needs [8, 9]. The environment, e.g., a homelike environment, including familiar music [10] and specific treatment such as the use of person-centered care [11] by personnel in SCU’s, appear to have a positive effect on the behavioural aspects of dementia [12, 13]. In the Netherlands, SCU’s for patients with dementia traditionally were institutionalized settings with shared bedrooms and large living rooms [14, 15]. In addition to these ‘regular’ SCU’s, small scale homelike SCU’s, where the group size is substantially smaller and the patients have private rooms are developed to meet the particular needs of patients with dementia more adequately. Small scale homelike SCU’s may vary in environmental design and philosophical concept with a corresponding specific approach. The environmental design can vary in (non-institutional) character of the units and the location of nursing stations [16]. The staff is specially trained in integrating meaningful activities around normal housekeeping, where the patients participate in activities such as cooking, shopping, cleaning and doing the laundry. Homelike elements, as for example, a kitchen or vegetable garden, are integrated into the living environments [17]. For an overview of patient characteristics between SCU’s and small scaled homelike SCU’s we refer to Verbeek et al. [18].

Compared to regular SCU’s, residents in small scale homelike SCU’s were found to need less support with activities of daily life [18, 19], to be more socially engaged [19], to show less agitation over time [20] and to need less psychotropic medication and physical restraints [20]. Furthermore, residents of small scale homelike SCU’s awarded higher scores to aspects of quality of life, showed less negative affect [18], better social relationships and were more engaged in activities [21].

Taking all these positive behavioural and emotional effects of living in a small scaled homelike SCU into consideration, the question arises whether the (decline of) cognitive functioning also differs between patients living at a regular SCU and at a small scale homelike SCU. Longitudinal research on cognitive functioning of patients with dementia, comparing small scale homelike SCU’s, regular SCU’s and non-SCU’s, however, are scarce.

Only two studies compared regular SCU’s with small scale homelike SCU’s. These studies showed neither significant differences over time in global cognitive functioning assessed with the Mini-Mental State Examination (MMSE) [19, 22] nor, more specific, in memory, as assessed by the Revised Memory and Behaviour Problems Checklist [19].

In the present study differences over time in specific cognitive functions of patients with dementia between regular SCU’s and small scale homelike SCU’s will be reported.

## Methods

We had the opportunity to investigate the effects of small scale homelike SCU’s on cognition in patients with moderate to severe dementia over time. The patients moved from a regular SCU consisting of units with 15 to 30 patients with bedrooms up to 4 patients to a small scaled homelike SCU with 7 to 8 patients per unit and single bedrooms. The control group consisted of patients who did not make this transfer but stayed at a regular SCU. Besides observational measures, we administered objective assessment instruments for specific cognitive domains as memory, executive function, language and praxis one month before and three and six months after the relocation.

Besides differences in the size of the units and single or shared bedrooms, another important difference is the amount of activities. Patients in the small scaled homelike SCU were more engaged in daily chores and did their own cooking and washing if possible, supervised by nurses, whereas the patients in the regular SCU got their meals from the institution kitchen and did not participate in household activities.

### Study design

The study is a longitudinal, quasi experimental field study with a treatment and control group.

### Participant characteristics

To determine sample size, a power analysis was performed using the statistical power analysis program G*power 3.1.7 [23], including a 2x2 repeated measures design, with one between subjects factor (home-like versus regular) and one within subjects factor (pre versus post), an alpha set at .05, a moderate effect size Cohen’s d = .30 (based on three studies on cognition in dementia in SCU’s compared with n-SCU’s [24–26]. This resulted in a total sample size of 111 subjects.

Inclusion criterion to participate in the study was a diagnosis of dementia reported in the medical file. 186 patients were assessed for eligibility of whom 145 consented at onset.

All patients suffered moderate to severe dementia and lived in regular SCU’s at two different locations of a health care institute in the Northern part of The Netherlands. The relocation of patients to the small scaled homelike SCU at one location was required due to organisational reasons; the building no longer met the requirements of the current healthcare standards.

During the study, the patients in the control group (*n* = 68) stayed at the same regular SCU with 20 to 30 patients per ward. The intervention group (*n* = 77) moved after baseline measurement from a regular SCU to a small scaled homelike SCU.

**Fig. 1:**
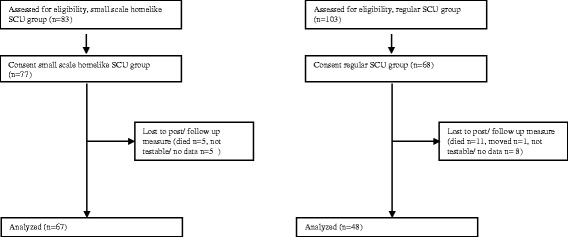
Flowchart of assessed groups

For a flowchart of the assessed patients, see Fig. 1.

Information about gender, age, global cognition, educational level, depression and subtypes of dementia are presented in Table 1.

**Table 1:** Demographic characteristics of the participants at baseline for both groups

	Small scale homelike SCU group *M (SD)*	Regular SCU group *M (SD)*	Test statistic	drop out small scale homelike SCU group *M (SD)*	drop out regular SCU group *M (SD)*
Sample size (*n*)	67	48		10	20
Gender	F 47, M 20	F 32, M 16	.158^a^ (n.s.)	F 6, M 4	F 17, M 3
Age (years)	83.27 (6.3)	82.88 (8.3)	.772^b^ (n.s.)	84,10 (5.1)	82,60 (6.1)
SMMSE	8.62 (6.5)	8.55 (6.3)	.961^b^ (n.s.)	13.00 (−^d^)	7.63 (7.5)
Depression	1.4 (1.2)	1.1 (0.9)	.272^b^ (n.s.)	0.00 (−^d^)	0.60 (0.6)
Education^c^	3.32 (1.4)	3.39 (1.3)	.782^b^ (n.s.)	2.86 (1.2)	3.10 (1.5)

^a^pearson chi square test, ^b^ *t*-test. (two-tailed), ^c^Conform Verhage [49], ^d^one participant

### Procedures

Due to capacity problems of the investigators it was not possible to assess both groups in the same year. Participants were recruited in the period February till May with one year difference between the small scale homelike SCU group and the regular SCU group. This way, we were able to assess both groups in the same months of the year and with the same intervals to avoid seasonal influences on mood and mood related cognitive effects.

The assessment took place in consultation with the caring nurses. We asked them to indicate the best moment of the day for each specific patient.

#### Intervention

The new wards were in the immediate vicinity of the old traditional nursing home and were all situated in one building. The provided health care and specific skills of personnel were the same in both conditions and remained largely the same during the course of the study. All personnel had been trained in handling and caring for patients with dementia. The content of the nine-hour training focussed on person centered care [11] and ethical aspects in the care for patients with dementia performed by external and internal trainers.

The experimenters assessed the patients with neuropsychological tests, while observational information from legal representatives and nursing personnel was obtained with specific questionnaires regarding observed cognitive abilities of the patients. Family members filled in the questionnaires at home and the nursing personnel filled in the questionnaires at the units. We composed a rather compact but diverse test battery with a maximum total examination time of one hour. Most patients completed the tests within the expected time on the basis of the instruction manual but some patients needed more sessions to complete the test battery. The experimenters were all trained master students of the psychology department of the University of Groningen. During all assessments a senior psychologist, not directly involved in the study, was available to support the students when necessary. None of the researchers have tested the same subject twice to avoid experimenter biases. All measures were assessed one month before relocation (baseline), 3 months after relocation (post-test) and 6 months after relocation (follow up test).

## Informed consent

The Ethical Committee of the department of Psychology of the University of Groningen, the Netherlands has approved the study (no. PPO-008-093). All legal representatives of the patients with a diagnosis of dementia received a letter with an explanation of the study. The legal representatives wrote an informed consent if they agreed that the patient could participate in the study. Besides this consent, before every assessment, the patient him/herself was verbally and non-verbally invited to assent. The investigators were instructed to stop the assessment if the patient showed resistance of any kind, verbally or non-verbally. The assessment would be continued at another moment in time or terminated definitely.

### Measures

#### Mood

Mood was assessed with the Dutch version [27] of the Geriatric Depression Scale-15 (GDS-15) [28], a self report assessment to identify depression in the geriatric population.

##### Global cognitive functioning

The Dutch version of the Standardized Mini-Mental State Examination [29–31] was used as a global measure of cognitive status. The SMMSE holds 19 questions and results in a maximum score of 30 points. The score was the number of correct answers. Patients scoring below 10 points are considered to be severely demented, between 10 and 19 moderately demented, between 20 and 26 mildly demented and a score above 26 is regarded as normal.

##### Specific cognitive domains

Five cognitive domains were assessed with various neuropsychological tests: (1) memory and learning, subdivided into (1a) verbal memory, (1b) memory for pictures, (1c) memory for faces, (2) language, subdivided into (2a) verbal fluency and (2b) naming of pictures, (3) executive functioning, (4) praxis abilities and (5) visual perception.

#### Verbal memory

With the Eight Word Verbal Memory Test of the Amsterdam Dementia Screening Test (ADS) [32] verbal memory was assessed. This test is validated for elderly patients with dementia. Immediate Recall (maximum score 40), Delayed Recall (maximum score 8) and Recognition (maximum score true positives 8) were assessed. The score was the number of correct reproductions.

#### Visual memory

Two subtests of the Rivermead Behavioural Memory Test (RBMT) [33] were used to assess visual memory; recognition of pictures (maximum score 10) and recognition of faces (maximum score 5). Outcome measure: number of correct recognitions.

#### Language

The shortened Boston Naming Test (BNT) [35, 36], assessing word finding, was used to asses language functioning. The shortened BNT consists of 29 items [37] and uses a scoring method based on the Aken Aphasia Test [38] with a maximal correct score of 29 points. Outcome measure: number of correct answers.

#### Praxis

The diagnostic test for apraxia of van Heugten [39-41] was used to assess different aspects of praxis; ideomotor and ideational praxis. Demonstration of item use and imitation of gestures and acts were examined (maximum score 90 points). Outcome measure: points obtained.

#### Executive functioning

The Trail Making Test A and B [42], a measure of executive control, The Category Fluency Task (naming as many animals as possible within one minute) from the Groningen Intelligence Test (GIT), measuring the skill and speed of searching through semantic memory [34] and a Clock Drawing test, tapping into a wide range of cognitive abilities including executive functioning, were used to assess aspects of executive functioning. The outcome measure ‘total correct sequenced rounds connected’ was used for the TMT-A (maximum score; 25) and TMT-B (maximum score; 25). The CLOX [43], spontaneous clock drawing (CLOX 1) and copy (CLOX 2), was used with a maximum score of 15 points. Outcome measure: points obtained.

#### Visual perception

For assessing figure recognition, a subtask of the GIT [34], namely Incomplete Drawings, was used (maximum score of 20). Outcome measure: number of correct interpretations of the incomplete drawing.

##### Observation questionnaire

*Observation of cognitive functions by nursing personnel.* Aspects of cognition were observed by nursing personnel with a behavioural observation scale for intramural psychogeriatry (GIP) [44]. This observation instrument is validated to judge social, cognitive and emotional behaviour of patients in nursing homes. The subscales Incoherent Behaviour (5 items), Memory Disturbance (7 items) and Disorientation (5 items) were used for this study. The nursing personnel had to indicate the frequency with which the problems occurred on a 4-point scale from never to (almost) always. Outcome measure: total of accumulated points.

*Observation of cognitive functions by representatives*. The Information Questionnaire on Cognitive Decline in the Elderly [45] is a screening tool, designed to report on cognitive decline in elderly people. A Dutch version of this IQCODE [46] was filled in three times by the legal representatives. The first time they judged the decline in cognition during the last six months before the start of the survey, the second time the decline between pre and post measurement and the third time between post and follow up measurement. The items of the IQCODE-N were weighted before analyzing; ‘much worse’ multiplied with −3, ‘slightly worse’ multiplied with −2, ‘not changed’ multiplied with 1, ‘slightly better’ multiplied with 2, and ‘much better’ multiplied with 3.

### Data analysis

Raw scores were used for analysis. For all dependent variables (neuropsychological test scores and observational measures), univariate analyses of covariance were performed with the baseline-score as covariate and the post or follow-up scores as dependent variable. The between subjects factor was type of SCU.

Besides raw scores, we analyzed the results on clusters of test measures. Therefore we standardized and summed related variables to identify possible tendencies within different domains.

For effect size (95 % CI), eta squared was used, of which .01–.05 is considered as small effect size, .06–.13 as moderate and .14 and higher as large [47]. We used SPSS version 18 to analyse the data.

## Results

### Baseline measurements

#### Demographics

At baseline, no significant differences (*p* < 0.05) were found in demographics, mood, cognition (Table 1) and type of dementia (Table 2) between both groups.

**Table 2:** Type of dementia of the participants

Type dementia	Small scale homelike SCU group *N* (%)	Regular SCU group *N* (%)
Dementia nos	18 (23)	26 (38)
Alzheimers’dementia	24 (31)	13 (19)
Vascular dementia	5 (7)	8 (12)
Mixed dementia	6 (8)	11 (16)
Lewy body dementia	1 (1)	1 (2)
Frontotemporal dementia	0 (0)	4 (6)
Other^a^	4 (5)	1 (2)

*Nos* not otherwise specified

^a^Parkinson dementia, alcohol dementia, Korsakov, semantic dementia, corticobasal degeneration

Neither were significant demographic differences (*p* < 0.05) found between the drop-outs of both groups (Table 1).

### Post-test

Data-analysis by means of ANCOVAs with the scores at the first measurement as covariate (see Table 3) resulted in only one significant difference (*p* < 0.05) with a large effect size (95 % CI), i.e., on recognition of pictures (RBMT); the small scale homelike SCU group showed a slight improvement over time, whereas the regular SCU group showed a small decline over time. Further, computing effect sizes in the pre-post comparison, a moderate effect size was found for recognition of faces (RBMT) and global cognitive decline (IQCODE-N) observed by representatives in favour of the small scale homelike SCU. One moderate effect size was found in favour of the regular SCU, namely Trailmaking B. The number of patients who completed this test however, was very small (*n* = 11).

**Table 3:** Pre-post-follow up values for both groups and differences between the groups at post test and follow-up test, controlled for pre test

							Effect group					
	Small scale homelike SCU M (SD)			Regular SCU M (SD)			Post			Follow up		
	Pre	Post	Follow up	Pre	Post	Follow up	*N*	*P*	*Ƞ* ^*2*^	*N*	*P (F)*	*Ƞ* ^*2*^
SMMSE^a^	8.7 (6.5)	9.1 (7.3)	9.0 (7.1)	8.37 (6.5)	7.9 (6.2)	8.9 (5.6)	64	.51	.01	54	.85	.00
8WT total^a^	8.6 (8.0)	9.0 (7.3)	9.7 (6.2)	8.0 (6.6)	9.3 (7.6)	9.7 (6.7)	37	.53	.01	32	.87	.00
8WT recall^a^	0.0 (0.2)	0.3 (1.1)	0.2 (0.8)	0.2 (0.6)	0.2 (0.9)	0.0 (0.0)	37	.55	.01	31	.33	.04
8WT recognition^a^	4.6 (2.7)	5.0 (2.7)	4.7 (2.9)	4.3 (2.6)	4.4 (2.9)	3.8 (2.8)	37	.67	.01	30	.59	.01
RBMT pictures^a^	11.2 (4.6)	13.2 (3.7)	12.4 (4.5)	12.8 (3.4)	11.3 (4.1)	11.1 (4.3)	45	.003	.19	36	.06	.10
RBMT faces^a^	5.7 (1.6)	6.2 (1.7)	6.0 (1.3)	6.5 (2.0)	5.8 (1.7)	5.4 (1.7)	42	.09	.07	30	.12	.09
Fluency^a^	7.7 (6.1)	7.5 (7.4)	7.0 (7.3)	6.4 (5.0)	7.2 (7.4)	8.2 (6.7)	50	.84	.00	40	.64	.01
BNT^a^	3.8 (4.0)	3.7 (4.5)	4.1 (4.5)	3.8 (4.5)	4.1 (6.1)	3.6 (5.4)	60	.61	.01	49	.49	.01
Praxis total^a^	56.7 (24.7)	52.0 (31.0)	59.1 (30.6)	60.0 (23.7)	51.0 (33.5)	60.3 (23.5)	43	.74	.00	33	.64	.01
TMT A^a^	16.5 (10.6)	15.8 (11.3)	13.7 (11.5)	13.3 (10.5)	12.7 (10.4)	15.6 (11.1)	31	.54	.02	25	.73	.00
TMT B^a^	4.5 (7.9)	3.9 (9.0)	4.5 (10.1)	0.5 (1.1)	1.7 (1.6)	1.5 (2.4)	12	.30	.12	10	.39	.11
Clox 1^a^	3.2 (4.3)	3.1 (3.8)	4.6 (4.7)	2.3 (3.3)	1.6 (2.0)	2.9 (2.6)	34	.34	.03	25	.52	.02
Clox 2^a^	4.5 (4.7)	4.6 (5.1)	6.7 (5.5)	5.4 (3.8)	5.8 (4.2)	7.1 (4.3)	32	.80	.00	25	.65	.01
GIT2 figure recogn.^a^	1.6 (2.3)	2.2 (2.7)	2.2 (3.0)	2.1 (4.2)	1.3 (1.6)	1.3 (1.1)	43	.17	.05	33	.06	.11
GDS15^b^	1.3 (1.2)	1.2 (1.0)	0.8 (0.7)	1.0 (0.8)	0.9 (0.9)	0.8 (0.7)	50	.62	.01	38	.88	.00
Incoherent behaviour^b^	9.1 (3.2)	8.8 (2.9)	8.3 (2.9)	8.8 (3.5)	9.0 (3.8)	9.1 (3.5)	90	.78	.00	89	.07	.04
Memory disturbance^b^	18.6 (4.3)	18.8 (4.4)	17.8 (4.8)	17.3 (4.9)	18.9 (4.8)	17.6 (4.6)	90	.22	.02	88	.65	.00
Disorientation^b^	9.0 (2.4)	9.4 (2.6)	9.1 (2.6)	9.3 (3.0)	10.9 (3.3)	9.5 (3.2)	90	.07	.04	88	.74	.00
IQCODE-N	−40.4 ((14.7)	5.5 (17.3)	4.3 (20.7)	−43.5 (9.3)	−5.4 (26.0)	−6.5 (25.1)	77	.03	.07	63	.06	.06

Ƞ^2^ = partial eta square, ^a^higher score = better performance, ^b^higher score = more impairment

For all other variables small or no effect sizes were found for the pre-post condition.

### Follow-up

In the pre-follow up comparison none of the test results showed a significant difference between the two groups (ANCOVA see Table 3).

A moderate effect size (95 % CI) in favour of the small scale homelike SCU was found for recognition of pictures (RBMT), recognition of faces (RBMT), figure recognition (GIT) and global cognitive decline observed by representatives (IQCODE-N). One moderate effect size was, again, found in favour of the regular SCU, namely Trailmaking B, again with a very small number of subjects (*n* = 9).

All other measures showed no or only small effect sizes in the pre-follow up comparison.

We used 18 different dependent variables. To reduce inflated type I errors, we used an alpha level of .05 to identify statistically significant effects. Applying the Bonferroni correction, with a modified *p*-value (*p* < 0.003), no significant *P* values were found between both groups.

### Clustered variables

The domains episodic memory (RBMT pictures, RBMT faces, Eight word test, Cronbachs *α* = .293), executive functions (Fluency, Clox 1 and 2, TMT A and B, Cronbachs *α* = .845) and global cognitive functioning (SMMSE, IQCODE, Cronbachs *α* = .307) showed one statistically significant difference (*p* < 0.05) for global cognitive functioning for the pre-post comparison in favour of the small scale homelike SCU.

A large effect size (95 % CI) for the post and follow up condition for the domain episodic memory and a moderate effect size for the domain global cognitive functioning was found in favour of the small scaled homelike SCU. A moderate effect size in favour of the regular SCU for both conditions was found for the domain executive functioning, both with a small number of analyzed subjects (*n* = 11, *n* = 9) (Table 4).

**Table 4:** Clustered z-scores pre-post-follow up values for both groups and differences between the groups at post test en follow-up test, controlled for pretest

							Comparisons					
	Small scale homelike SCU M (SD)			Regular SCU M (SD)			Pre - post			Pre - follow up		
	Pre	Post	Follow up	Pre	Post	Follow up	*N*	*P*	*Ƞ* ^*2*^	*N*	*P*	*Ƞ* ^*2*^
Episodic memory	−0.3 (2.2)	0.4 (2.2)	0.5 (2.3)	0.4 (2.1)	−0.1 (2.7)	−0.1 (2.3)	33	.51	.42	27	.47	.43
Executive functions	44.3 (28.6)	49.6 (31.8)	55.4 (34.1)	35.7 (17.9)	44.4 (22.2)	45.8 (17.0)	11	.76	.83	8	.62	.90
Global cognitive functioning	−28.7 (19.2)	12.8 (17.2)	11.2 (22.7)	−35.5 (12.1)	−1.4 (29.5)	−3.5 (28.5)	53	.03	.07	36	.09	.10

## Discussion and conclusion

In this study we examined possible beneficial effects of small scale homelike SCU compared with a regular SCU on specific cognitive functions of patients with dementia.

Although no significant differences between both groups over a period of six months were found, the majority of effect sizes suggest that living at a small scale homelike SCU might have a beneficial influence on cognitive functioning, in particular visual memory and recognition of pictures. The effect sizes of the domains episodic memory and executive functioning are large respectively .43 and .90. The observations of the legal representatives show the same tendency, indicating some convergent validity.

Our results correspond with results described in earlier studies comparing regular SCU’s with small scale homelike SCU’s, using global cognitive screening instruments [19, 22]. The subjects tested in those studies were mildly to moderately impaired patients with Alzheimer’s disease with mean MMSE scores ranging from 10.3–15.4 [19] and from 11.1–11.3 [22]. In our study, the patients suffered from moderate to severe dementia and had a mean MMSE score of approximately 8.5. Presumably the severity of the dementia in our study lowered the chance of finding a positive effect of living at a small scale unit on cognitive functions. Moreover, it appeared that the majority of our patients were not able to complete *all* tasks, resulting in small sample sizes. Further, the heterogeneity of the different types of dementia within the groups could have clouded the effects.

However, even given these limitations, one might argue that every tendency in the direction of better performance (objectively assessed or subjectively observed) in the small scale homelike group is a worthwhile finding. Beforehand one could have argued that the process of relocation would cause a profound disturbance in the daily life and behaviour of the demented patients, with an accompanying decline in cognitive functioning. As a Dutch saying suggests: “never move an old tree”. This study shows no effects of turmoil whatsoever. The patients moving to the small scaled homelike SCU even appear to improve their performance, although to a modest degree, while the patients who did not undergo an extensive change in environment seem to remain at the same level or deteriorate (again to a modest degree), as might be expected from patients with a moderate to severe dementia.

Literature search shows scarce quality of life research in patients with dementia. In two studies no differences over time in small scale homelike SCU’s compared with regular SCU’s assessed with global instruments were found [18, 20] but specific aspects of quality of life as positive affect and social relations were more frequent in a small scale homelike SCU compared with a regular SCU [21]. In our opinion, more specific research on quality of life can reveal the positive effects of small scaled homelike living.

The present study is part of a larger study. Results about behaviour, medication use, quality of life and mood will be presented in future articles.

### Limitations

A limitation of our study is non-randomization of the subjects to the conditions. However, ethical aspects prohibit randomization and we believe that our study design is preferable to cross sectional studies. In our research, the sample size is limited and therefore the findings should be considered with caution. Research with a larger sample size is recommended.

Because of restricted permission of examination time by the ethical committee, we composed a rather compact but diverse test battery to allow for the assessment of at least the most important cognitive symptoms/criteria with a total maximum examination time of one hour. A negative consequence of this procedure might be that some areas of cognitive functioning as intelligence and working memory have not been studied. On the other hand we feel that a more extensive assessment is hardly possible in this seriously ill patient population.

The groups were assessed in two consecutive years due to researcher capacity problems. Because of the fact there were no baseline differences between both groups we do not expect an influence of this year difference.

Although we used the most accessible and easy tests for this population of patients, there are bottom effects in testing patients with severe dementia. To compensate for this limitation, we added behaviour observation lists for nursing personnel and representatives about cognition. These results neither show significant differences between groups. The observation lists have a restricted response range and they are limited in sensitivity. Generally, observation lists show limited correlation with more direct methods of collecting data [48].

### Conclusion

The findings of the present study suggest that there is no difference between two types of care facilities for demented residents concerning (a decline in) global and specific cognitive functions over a certain period of time. Longitudinal research with more subjects over a longer period of time is recommended to explore the specific aspects of these care facilities in relation to cognition.

## Abbreviations

SCU: special care unit
CI: confidence interval
BPSD: behavioral and psychological symptoms of dementia
SMMSE: standardized mini-mental state examination
GDS-15: geriatric depression scale – 15 items
ADS: Amsterdam dementia screening test
RBMT: Rivermead behavioural memory test
BNT: Boston naming test
GIT: Groningen intelligence test
TMT: trail making test
GIP: behavioural observation scale for intramural psychogeriatry
IQCODE: information questionnaire on cognitive decline in the elderly
SPSS: statistical package for the social sciences
ANCOVA: analysis of covariance
